# Supplementary material for: Multi-tissue DNA methylation age predictor in mouse
Source: Genome Biol. 2017 Apr 11;18:68. doi: 10.1186/s13059-017-1203-5 (PMC5389178; doi:10.1186/s13059-017-1203-5)
Supplement: Supplementary file 2 — Characterisation of ageing associated methylation changes at discrete CpGs, related to Fig. 1. (PDF 974 kb) [file 13059_2017_1203_MOESM2_ESM.pdf]

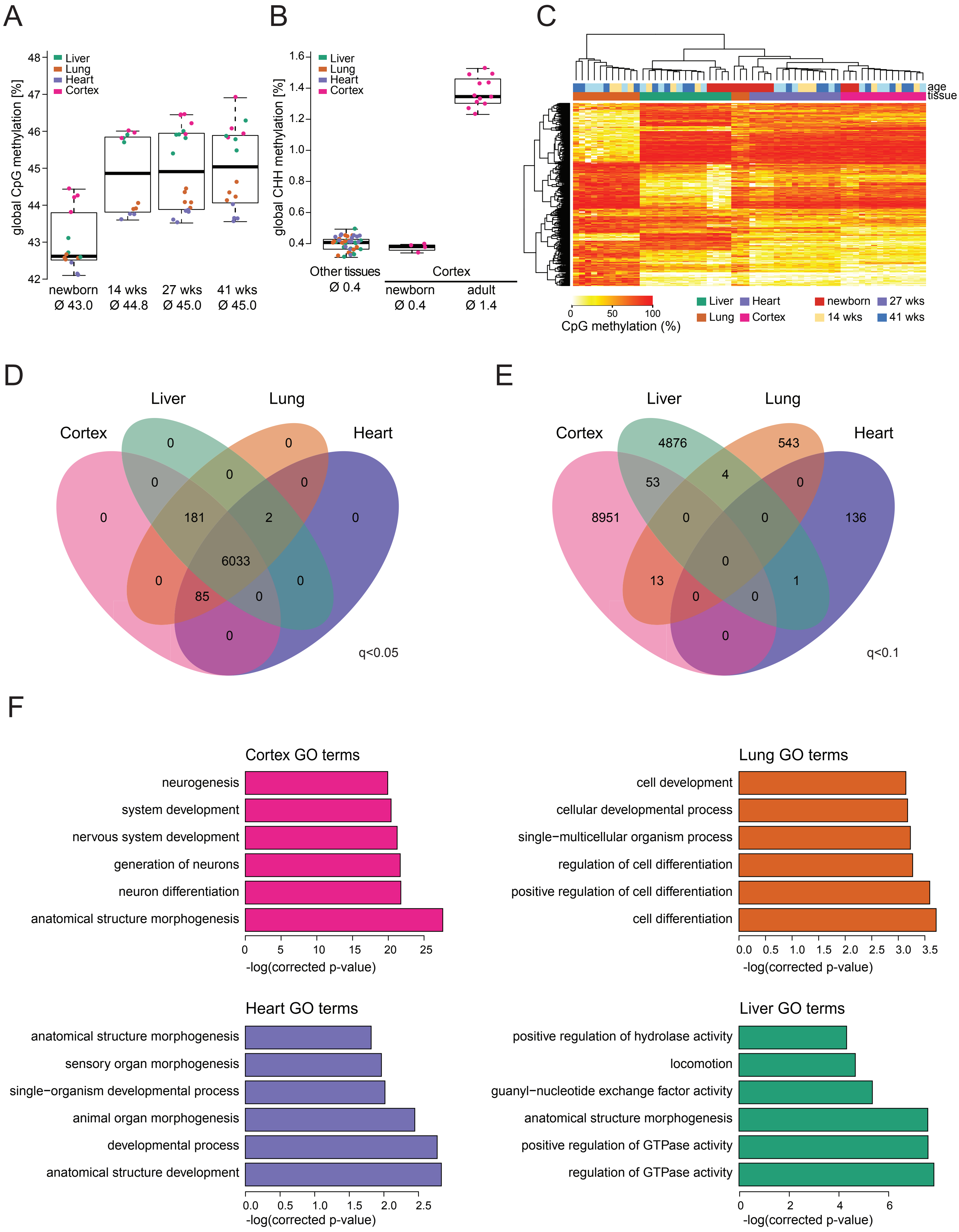

Additional File 2: Characterisation of ageing associated methylation changes at discrete CpGs, related to Figure 1

Additional File 2: Characterisation of ageing associated methylation changes at discrete CpGs, related to Figure 1

(A) Global CpG DNA methylation levels (%) in different tissues and at different ages. Samples are ordered by age (weeks). Mean CpG methylation levels were calculated for each sample. The colour highlights the tissue that each sample was derived from.

(B) Global CHH methylation levels (%) in different tissues and at different ages. Samples were grouped into "Other tissues" (containing heart, liver and lung samples of all four ages) and "Cortex", which was further subdivided into newborn (<1wk) and adult samples (14 weeks, 27 weeks and 41 weeks).

(C) Tissue clustering of the samples from different tissues and ages. Hierarchical clustering was performed using Manhattan distance. Age and tissue type of each sample is highlighted using column side colouring.

(D) Venn diagram of tissue-independent age-associated Spearman correlations with a corrected p-value (q-value) of <0.05. Shown is the overlap between the four tissues.

(E) Venn-diagram of tissue-specific age-correlations. The Spearman correlations were defined for each tissue with a corrected p-value cut-off of <0.1. Shown is the overlap between the four tissues.

(F) The top 6 GO terms associated with the tissue-specific correlations found for each tissue is presented.
