# Supplementary material for: Multi-tissue DNA methylation age predictor in mouse
Source: Genome Biol. 2017 Apr 11;18:68. doi: 10.1186/s13059-017-1203-5 (PMC5389178; doi:10.1186/s13059-017-1203-5)
Supplement: Supplementary file 3 — Characterisation of ageing associated methylation changes at discrete CpGs excluding newborn samples, related to Fig. 1. (PDF 1117 kb) [file 13059_2017_1203_MOESM3_ESM.pdf]

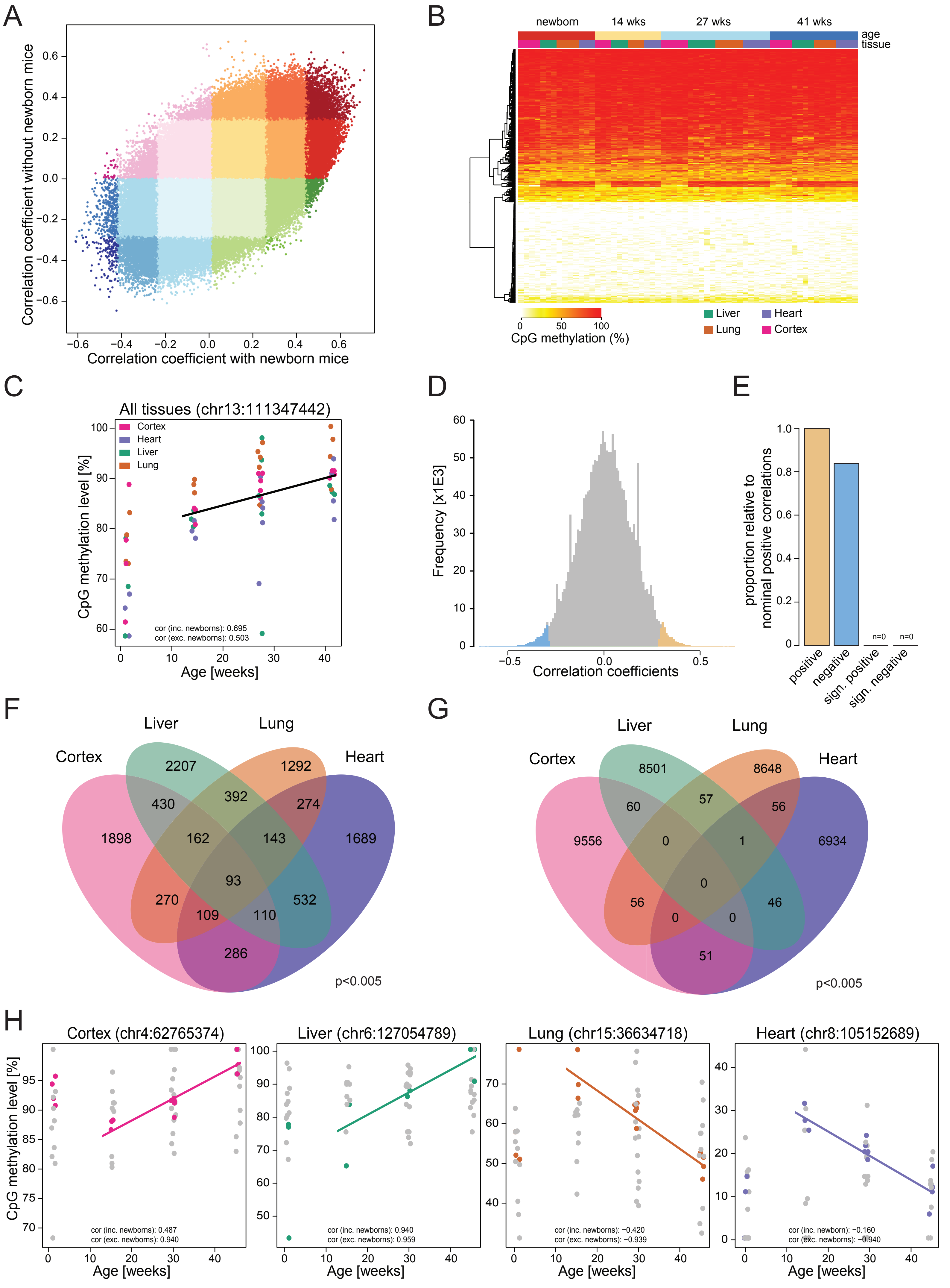

Additional File 3: Characterisation of ageing associated methylation changes at discrete CpGs excluding newborn samples, related to Figure 1

Additional File 3: Characterisation of ageing associated methylation changes at discrete CpGs excluding newborn samples, related to Figure 1.

(A) Scatterplot of conservation of Spearman correlation estimates with and without newborn samples. Correlations that are both negative or positive are shown in blue or red respectively. Correlations that differ in directionality with newborn-containing correlations being positive or negative are shown in green or pink respectively. Significance of correlation is emphasised by shading.

(B) Heatmap of the top 500 tissue independent age-associated correlations excluding newborns. Highlighted are ages and tissues, CG sites were clustered by Euclidean distance.

(C) Single CG sites within the genome are correlated with age excluding newborn samples from the analysis. Shown is an example site: chr13:111347442, Spearman correlations are detailed; inclusive and exclusive of newborn samples. Tissues are highlighted by colour. Jitter is for aesthetic purposes only.

(D) Overview of Spearman correlations calculated over all tissues excluding newborn samples. Distribution of correlation estimates are shown in the histogram. Nominal correlations are highlighted ($p<0.05$) in light orange (positive) and light blue (negative). There are no significant correlations, when newborn samples are excluded from the Spearman correlation.

(E) Proportionate number of correlations are shown in the barplot relative to nominally significant positive Spearman correlations. Nominal correlations are highlighted ($p<0.05$) in light orange (positive) and light blue (negative). There are no significant correlations, when newborn samples are excluded from the Spearman correlation.

(F) Venn diagram of tissue-independent age-associated Spearman correlations with a p-value cut-off of <0.005. Shown is the overlap between the four tissues.

(G) Venn-diagram of tissue-specific age-associated Spearman correlations. The Spearman correlations were defined for each tissue with a p-value cut-off of <0.005. Shown is the overlap between the four tissues.

(H) Tissue-specific Spearman correlations with age excluding newborn samples. An example is provided of a tissue-specific correlation with age in cortex, liver, lung and heart. Correlations for these CG sites are provided for all tissues combined and for the tissue in question. Jitter is for aesthetic purposes only.
