# Supplementary material for: Multi-tissue DNA methylation age predictor in mouse
Source: Genome Biol. 2017 Apr 11;18:68. doi: 10.1186/s13059-017-1203-5 (PMC5389178; doi:10.1186/s13059-017-1203-5)
Supplement: Supplementary file 5 — Properties of the mouse epigenetic clock sites, related to Fig. 2. (PDF 1310 kb) [file 13059_2017_1203_MOESM5_ESM.pdf]

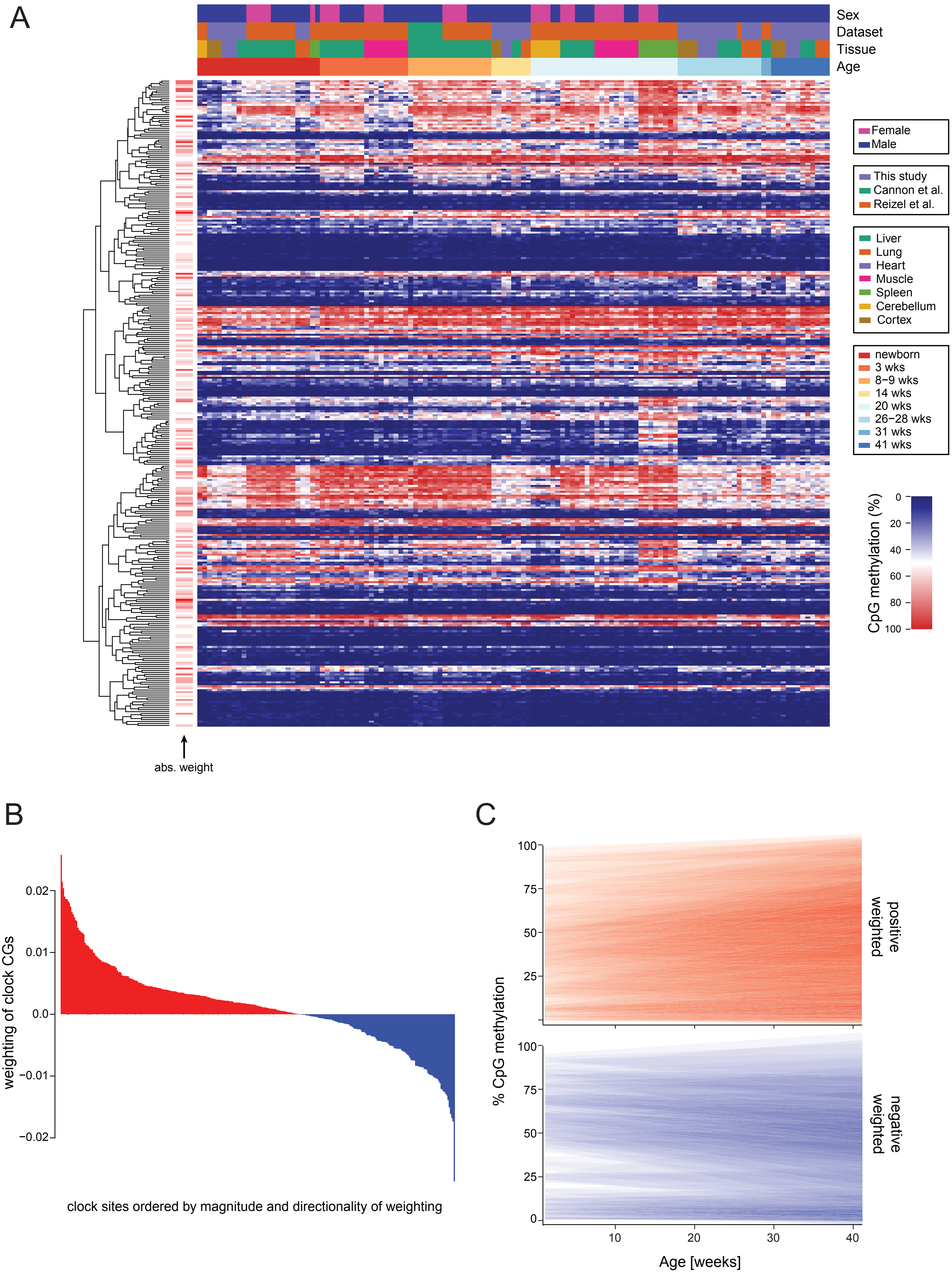

Additional File 5: Properties of the mouse epigenetic clock sites, related to Figure 2

Additional File 5: Properties of the mouse epigenetic clock sites, related to Figure 2.

(A) Heatmap showing the methylation level of the clock sites. Highlighted in the column side bars are: sex (upper side bar: male and female shown in pink and blue respectively), dataset (upper-middle side bar: Babraham, Cannon and Reizel are shown in purple, green and orange respectively), tissue (lower-middle side bar: liver, lung, heart, muscle, spleen, cerebellum and cortex are shown in green, orange, purple, pink, yellow-green, mustard, and brown), and age (lower side bar: ages are shown across a colour spectrum from red (<1wk) to blue (41wk)). The clock sites within the heatmap are clustered on Euclidean distance and the samples are ordered principally by age but followed by tissue, dataset and sex.

(B) Barplot representation of the weighting and directionality of the clock sites. Sites were ordered highest to lowest. Positively weighted sites are shown in red and negatively weighted in blue.

(C) Regression plot showing overall directionality of methylation changes at the clock sites. Negatively weighted sites are shown in blue and positively weighted sites in red. The initial width of each regression line was determined by the standard deviation at newborn age and the final width by the standard deviation at 41 weeks of age.
