# Supplementary material for: Multi-tissue DNA methylation age predictor in mouse
Source: Genome Biol. 2017 Apr 11;18:68. doi: 10.1186/s13059-017-1203-5 (PMC5389178; doi:10.1186/s13059-017-1203-5)
Supplement: Supplementary file 7 — Additional detail on the mouse epigenetic clock, related to Fig. 2. (PDF 152 kb) [file 13059_2017_1203_MOESM7_ESM.pdf]

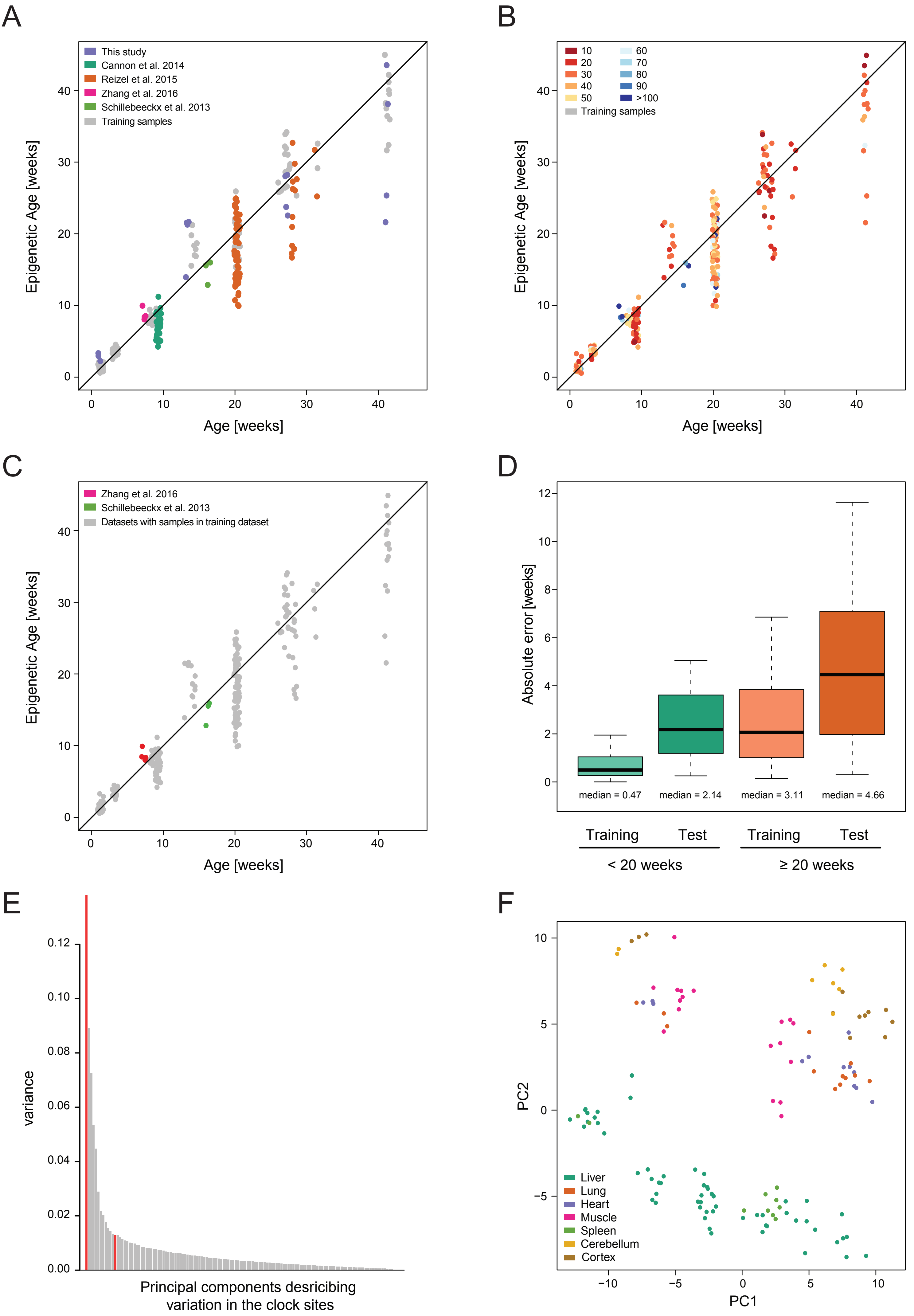

Additional File 7: Additional detail on the mouse epigenetic clock, related to Figure 2.

Additional File 7: Additional detail on the mouse epigenetic clock, related to Figure 2.
(A) Prediction of age coloured by their dataset of origin. The training data is shown in the background in grey.
(B) Average coverage of the prediction sites in the test samples. The training data is shown in the background in grey.
(C) Prediction of age in 2 unobserved datasets [31,32]. The training data is shown in the background in grey.
(D) Absolute error of the model as depicted per age group, young (defined as below 20 weeks of age) and old (defined as 20 weeks of age or older) in the test data and training data. The MAEs are displayed for each boxplot.
(E) Percentage of explained variation of the principal component analysis on the clock sites, components 1, and 13 are nominally significant related to age (shown in red).
(F) Principal component analysis of the first and second component as derived from the PCA on the clock sites in the training samples; coloured by tissue.
