# Supplementary material for: Multi-tissue DNA methylation age predictor in mouse
Source: Genome Biol. 2017 Apr 11;18:68. doi: 10.1186/s13059-017-1203-5 (PMC5389178; doi:10.1186/s13059-017-1203-5)
Supplement: Supplementary file 8 — Information on the datasets properties of the clock sites, related to Fig. 2. (PDF 153 kb) [file 13059_2017_1203_MOESM8_ESM.pdf]

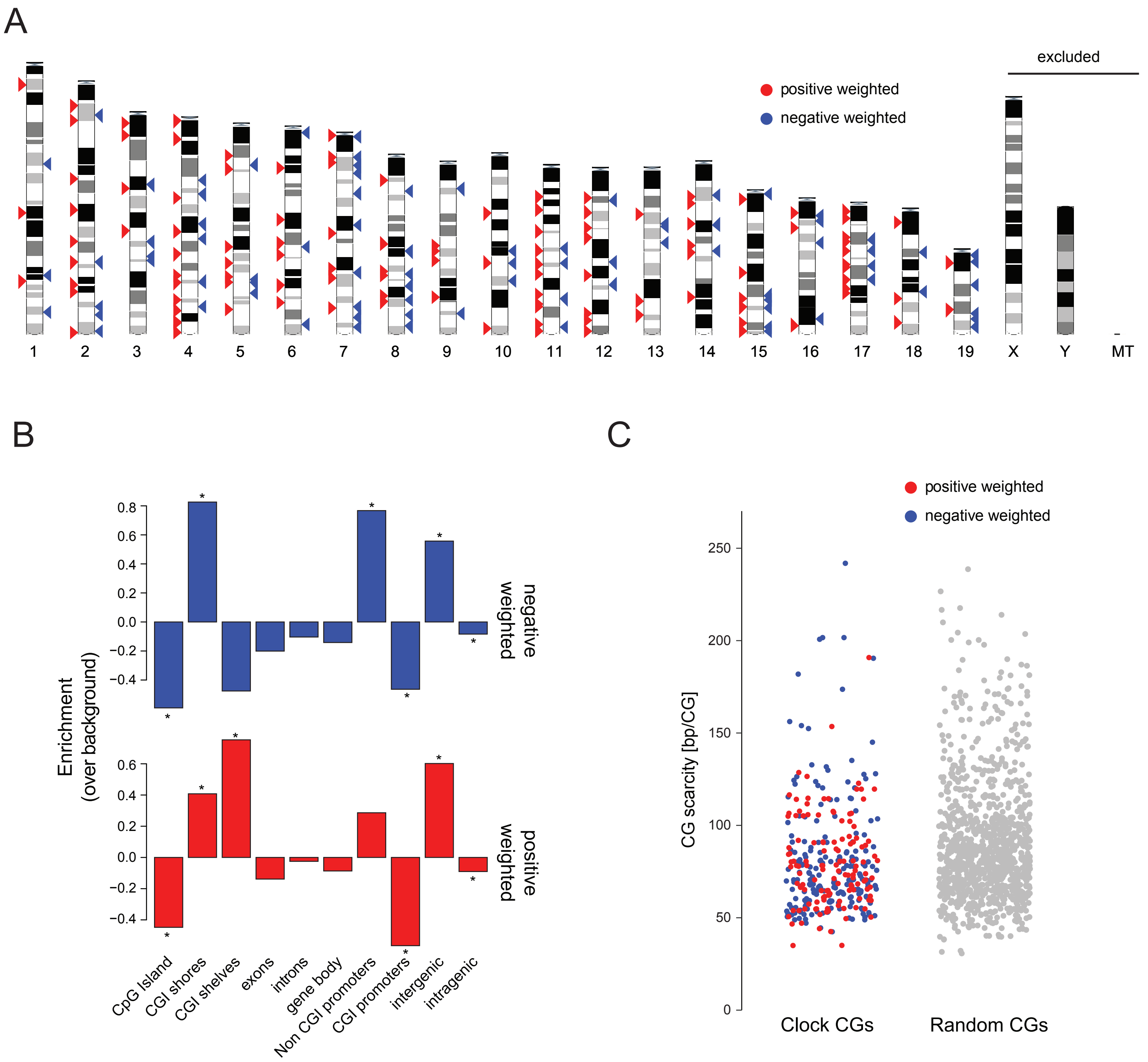

Additional File 8: Information on the datasets properties of the clock sites, related to Figure 2

(A) Ideogram showing the location of the clock sites on the mouse genome. Directionality is as indicated in the legend, with positively weighted sites displayed in by red arrows and negatively weighted sites by blue arrows.

(B) Enrichment of genomic features in the clock sites. Directionality of weighting displayed (red for positive weighting and blue for negative weighting). Tested using binomial test; *: p-value<0.05.

(C) CG scarcity of the clock sites compared to a random set of CG sites, coloured by directionality of weighting of the probes in the model.
