# Supplementary material for: Multi-tissue DNA methylation age predictor in mouse
Source: Genome Biol. 2017 Apr 11;18:68. doi: 10.1186/s13059-017-1203-5 (PMC5389178; doi:10.1186/s13059-017-1203-5)
Supplement: Supplementary file 9 — Testing age relation of the human clock sites in the mouse, related to Fig. 3. (PDF 538 kb) [file 13059_2017_1203_MOESM9_ESM.pdf]

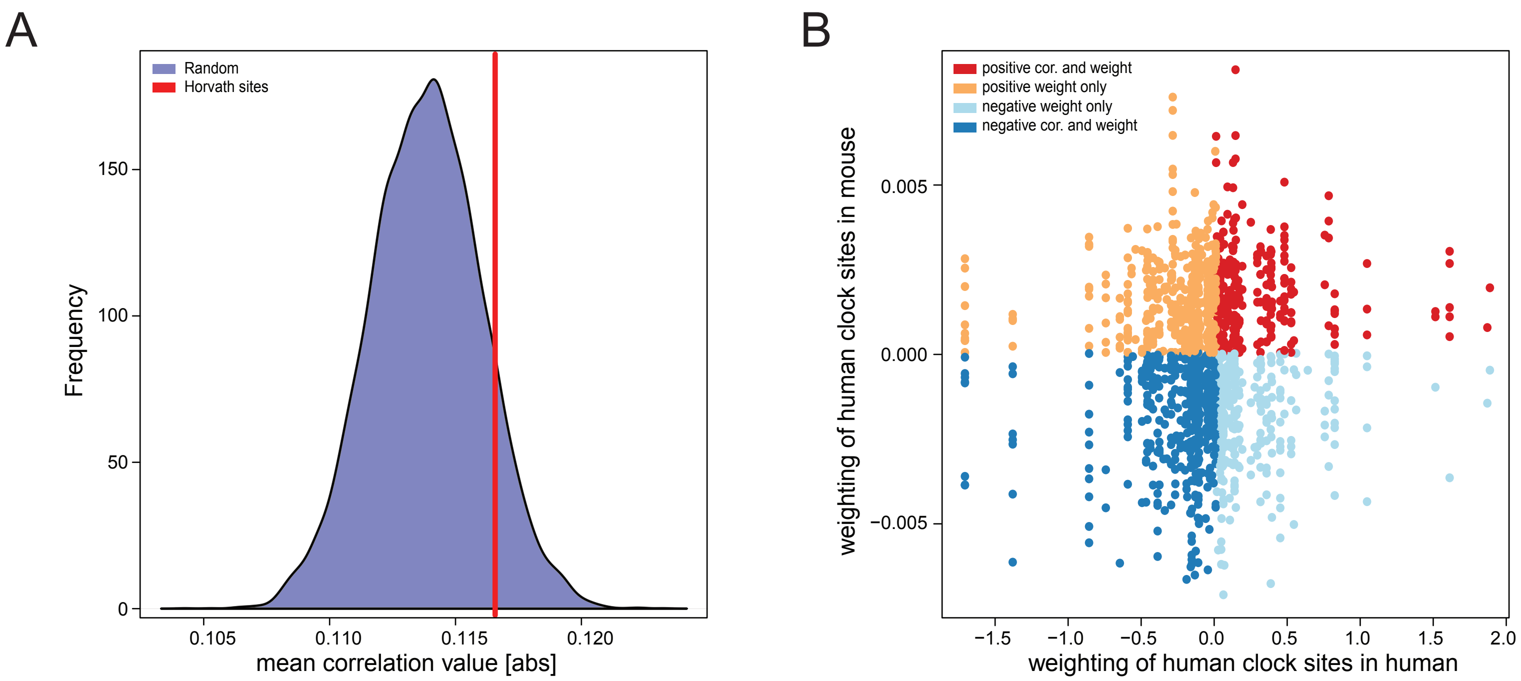

Additional File 9: Testing age relation of the human clock sites in the mouse, related to Figure 3.
(A) Average absolute age correlation of the 175 Horvath clock regions in mouse (red line) and the average absolute age distribution of one thousand randomly picked sets of 175 regions (blue).
(B) Comparison of the weighting of the human clock sites in the Horvath clock [6] with the weighting of the corresponding regions in the age prediction model generated using the Horvath clock regions in mouse.
