# Supplementary material for: Multi-tissue DNA methylation age predictor in mouse
Source: Genome Biol. 2017 Apr 11;18:68. doi: 10.1186/s13059-017-1203-5 (PMC5389178; doi:10.1186/s13059-017-1203-5)
Supplement: Supplementary file 10 — Predicted age of test samples as coloured by sex, related to Fig. 4. (PDF 95 kb) [file 13059_2017_1203_MOESM10_ESM.pdf]

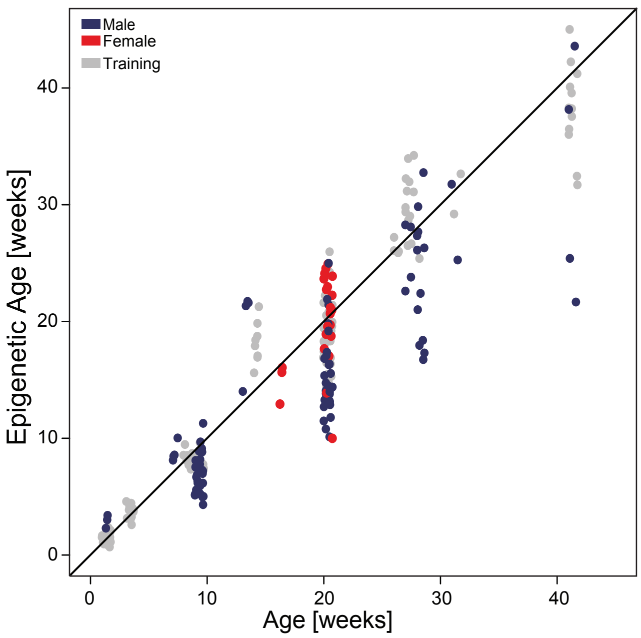

Additional File 10: Predicted age of test samples as coloured by sex, related to Figure 4. The training data is shown in the background in grey.
